# *Helicosporidium* sp. infection in a California kingsnake (*Lampropeltis californiae*): Spillover of a pathogen of invertebrates to a vertebrate host

**DOI:** 10.1177/03009858241259179

**Published:** 2024-06-16

**Authors:** Javier Asin, April L. Childress, Eva Dervas, Michael M. Garner, Francisco A. Uzal, James F. X. Wellehan, Eileen E. Henderson, Anibal G. Armien

**Affiliations:** 1 University of California, Davis, San Bernardino, CA; 2 University of Florida, Gainesville, FL; 3 University of Zürich, Zürich, Switzerland; 4 Northwest ZooPath, Monroe, WA; 5 University of California, Davis, Davis, CA

**Keywords:** algae, *Helicosporidium*, snake

## Abstract

*Helicosporidium* is a genus of nonphotosynthetic, green algae in the family *Chlorellaceae*, closely related to *Prototheca*. It is a known pathogen of invertebrates, and its occurrence in vertebrates has not been documented. A captive, 10-month-old, male, albino California kingsnake (*Lampropeltis californiae*) was submitted for necropsy. Gross examination revealed hemorrhagic laryngitis and a red mottled liver. Histologically, intravascular, intramonocytic/macrophagic and extracellular, eukaryotic organisms were observed in all tissues. These organisms stained positive with Grocott-Gomori methenamine silver and periodic acid-Schiff and were variably acid-fast and gram-positive. Ultrastructural analysis revealed approximately 4 µm vegetative multiplication forms and cysts with 3 parallel ovoid cells and a helically coiled filamentous cell. A polymerase chain reaction with primers targeting *Prototheca*, amplicon sequencing, and Bayesian phylogenetic analysis confirmed it clustered within *Helicosporidium* sp. with 100% posterior probability. The genus *Helicosporidium* was found to nest within the genus *Prototheca*, forming a clade with *Prototheca wickerhamii* with 80% posterior probability.

*Helicosporidium* sp. is a nonphotosynthetic green alga in the phylum Chlorophyta, class Trebouxiophyceae, order Chlorellales, and family *Chlorellaceae*, and is a pathogen of invertebrate hosts.^15^ Initially described as a protist or a fungus,^6,18^ recent phylogenetic studies identified the genus as algae closely related to *Prototheca*.^16,17^ In the initial reports, a single species, *Helicosporidium parasiticum*, was recognized.^6,18^ Later, the designation *Helicosporidium* sp. (ie, with no species name) was preferred,^20^ as species diversity is not well understood.^15^

*Helicosporidium* sp. has 2 distinct stages: a vegetative multiplication form containing 2, 4, or 8 daughter cells that divide through autosporulation, which is similar to other *Chlorellaceae*, including *Prototheca* spp.,^4,14^ and a cyst form, comprising 3 parallelly stacked ovoid cells surrounded by a helically coiled filamentous cell.^15^ The cyst is considered unique to, and diagnostic for, *Helicosporidium* sp. in invertebrate pathology and is best identified through transmission electron microscopy.^3,20^ Descriptions of *Helicosporidium* sp. in tissues using routine histochemical techniques are scarce in the literature.

Most reports of *Helicosporidium* sp. infection involve insect hosts, including several members of the orders Lepidoptera, Coleoptera, and Diptera.^15^ Infections in other invertebrate, noninsect hosts, such as mites or trematodes, have been occasionally described,^15^ but information on those species is limited. To our knowledge, infection by this genus has never been documented in a vertebrate host. We describe a case of *Helicosporidium* sp. infection in a California kingsnake (*Lampropeltis californiae*).

The carcass of a captive, 10-month-old, male, albino California kingsnake was submitted for necropsy to the San Bernardino branch of the California Animal Health and Food Safety laboratory system (University of California, Davis) in June 2021. The snake had been found dead in its cage without prior signs of illness. The animal had been purchased 9 months before death, was always housed individually, and was being fed neonatal (pinky) mice once a week. Grossly, there was a focus of hemorrhage in the larynx, and the liver was faintly mottled red and mildly enlarged (Fig. 1a).

**Figure 1.**
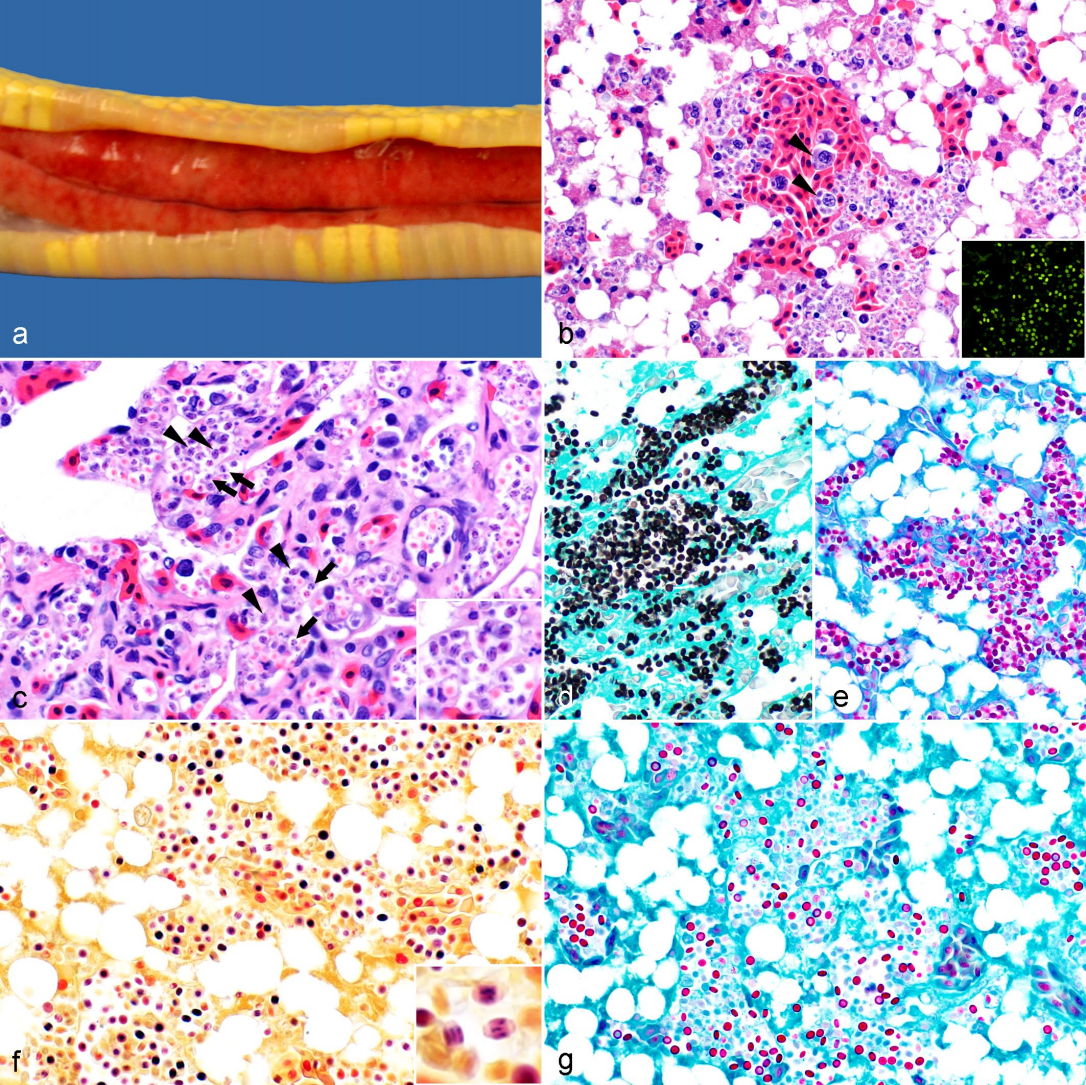
Gross and microscopic findings in a California kingsnake (*Lampropeltis californiae*) infected with *Helicosporidium* sp. (a) There is faint red mottling in the liver and mild hepatomegaly. (b) Liver. Abundant, extracellular and intramonocytic/macrophagic (arrowheads) vegetative forms and cysts; diffuse hepatocellular lipidosis. Hematoxylin and eosin (HE). Inset: The organisms display green autofluorescence. (c) Lung. The same organisms are observed packing the pulmonary vessels. Vegetative cells (arrows) display internal, amorphous basophilic structures (presumably daughter cells) occasionally separated by fine clear septa. Mature cysts (arrowheads) have 3 parallel, basophilic, stick-like ovoid cells. HE. Inset: Higher magnification of cysts and vegetative cells. (d-g) Liver. (d) All forms are positive with Grocott-Gomori methenamine silver and (e) periodic acid-Schiff stains. (f) The internal structures are gram-positive. Hucker-Conn Gram stain. Inset: Gram stain highlights the 3 ovoid cells in the cysts. (g) The wall of a subset of the forms (presumably cysts) is acid-fast. Ziehl-Neelsen.

Samples of lung, liver, larynx, esophagus, trachea, kidney, adrenal gland, testis, small intestine, colon, stomach, brain, spinal cord, thyroid gland, skeletal muscle, skin, bone marrow, and heart were fixed in 10% neutral-buffered formalin for 24 hours, embedded in paraffin, and processed to produce 4 µm thick, hematoxylin and eosin–stained sections. These sections were examined with light and fluorescent microscopes. Selected tissue sections were stained with periodic acid-Schiff, Grocott-Gomori methenamine silver, Hucker-Conn Gram, and Ziehl-Neelsen stains. Aerobic cultures of the pharynx and liver, and *Salmonella* sp. enrichment culture of the liver, were performed. In addition, a mineral screen, including arsenic, cadmium, copper, iron, lead, manganese, mercury, molybdenum, zinc, and selenium measurements, was conducted in liver.

Histologically, there were myriad intravascular, extra- and intracellular (within the cytoplasm of monocytes/macrophages), approximately 3 to 4 µm, discoid to oval eukaryotic organisms in all examined tissues, being particularly numerous in the liver, lungs, kidneys, and heart (Fig. 1b, c). The organisms had green and blue autofluorescence. Most of the forms had a <1 µm wall and stained light orange to faintly eosinophilic in the center; approximately half of them had oval to amorphous basophilic structures in the center that occasionally appeared to be separated by faint, clear septa, and approximately the other half had 3 central, parallel, basophilic, stick-like structures (Fig. 1c). All forms stained positive with periodic acid-Schiff and Grocott-Gomori methenamine silver stains, which obscured the internal structures (Fig. 1d, e). The internal structures, including the 3 parallel, stick-like structures, were gram-positive (Fig. 1f). The wall of a subset of the forms was acid-fast (Fig. 1g).

There was minimal granulocytic and lymphohistiocytic inflammation associated with the organisms and multiple foci of parenchymal drop-out in the liver, which also displayed diffuse lipidosis (Fig. 1b). In addition, there was necrohemorrhagic laryngitis and focally extensive necrohemorrhagic colitis with intralesional coccobacilli. Gram-negative coccobacilli were observed within the vessels of most of the organs examined. *Aeromonas veronii*, *Citrobacter freundii*, *Salmonella* sp. Group C2, and mixed flora were isolated from the liver. *Enterococcus faecalis*, *Vagococcus fluvialis*, *C. freundii*, *Morganella morganii*, and mixed flora were isolated from the larynx. The hepatic iron concentration was 100 ppm, slightly below the expected range for snakes (130–830 ppm; based on a small internal data set [*n* = 7]); the rest of the minerals were within acceptable ranges.

Transmission electron microscopy was performed on formalin-fixed, paraffin-embedded liver, lung, and heart as described.^14^ Briefly, cubic 1 to 2 mm^3^ sections were deparaffinized and post-fixed in 0.1 M cacodylate-buffered 2.5% glutaraldehyde and 1% osmium tetroxide (Electron Microscopy Sciences, Hatfield, Pennsylvania). Tissue fragments were embedded in resin in an automatic tissue processor, and 60 to 90 nm ultrathin sections were stained with toluidine blue, examined to identify the regions of interest, transferred to a copper grid, and examined with a JEOL 1400 transmission electron microscope (JEOL Ltd., Tokyo, Japan).

Owing to formalin fixation and paraffin embedding, moderate to severe processing artifacts affected the ultrastructure of the eukaryotic organism. Nevertheless, different forms could be identified. There were approximately 3.8 to 4.0 µm in diameter vegetative multiplication cells with 1 to 7 daughter cells visible (Fig. 2a); the vegetative cells had a nucleus, mitochondria, rough endoplasmic reticulum, polyribosomes, and Golgi apparatus. There was no evident chloroplast. These findings, particularly the absence of chloroplast and the pattern of autosporulation, suggested that this organism was an achlorophyllous alga of the family *Chlorellaceae*.^4,14^ In addition, there were approximately 3.9 µm cysts with three, 2 to 2.1 × 1 to 1.2 µm, parallelly stacked ovoid cells surrounded by a filamentous cell (Fig. 2b, c). The vegetative cells and cysts had a 0.06- to 0.1-µm multilayered wall and were surrounded by frequently fragmented pellicles with curled borders (Fig. 2b, inset). The mature cyst was considered compatible with *Helicosporidium sp.*^3,20^ Neither *Prototheca* sp. nor other members of the *Chlorellaceae* have been reported to develop this form, whose observation prompted additional molecular studies. A *Helicosporidium*-specific polymerase chain reaction was not available; therefore, an assay targeting *Prototheca* was performed.

**Figure 2.**
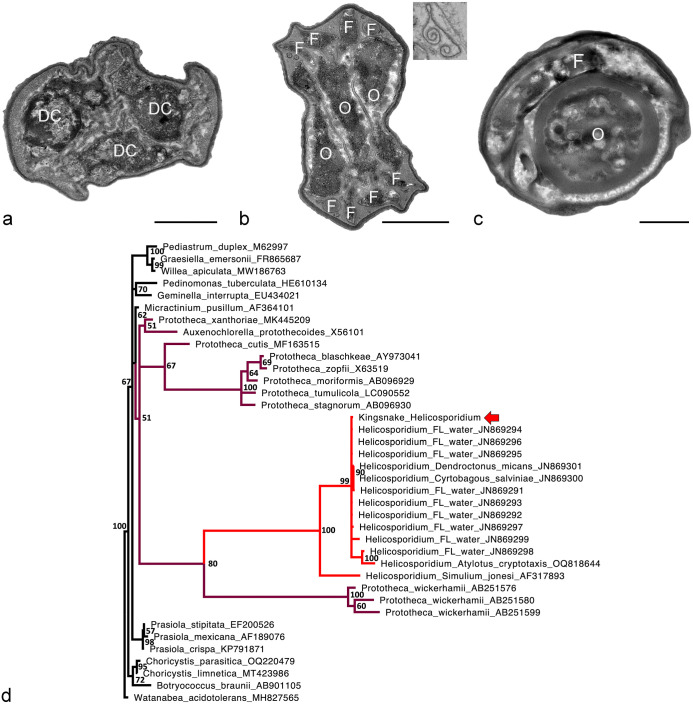
(a-c) Ultrastructural features of the different forms of *Helicosporidium* sp. observed in a California kingsnake (*Lampropeltis californiae*). There is moderate artifactual shrinkage due to formalin fixation and paraffin embedding prior to processing. Liver. Transmission electron microscopy. Bars = 1 µm. (a) Vegetative cell with 3 visible daughter cells (DC). (b) Cyst with 3 ovoid cells (O) surrounded by a coiled filamentous cell (F) with cross-sections visible in the poles. Inset: Fragment of a pellicle with curled borders. (c) Cyst with a visible ovoid cell (O) and a sagittal section of the filamentous cell (F) coiling around it. (d) Bayesian phylogenetic tree of MAFFT nucleotide alignments of selected Chlorophyta (GenBank accession numbers given after names) made using MrBayes 3.2.7a with a general time reversible model, gamma distributed rate variation, and a proportion of invariant sites; *Watanabea acidotolerans* was designated as the outgroup. The *Helicosporidium* sp. from a California kingsnake (red arrow; GenBank OR913728) clusters within *Helicosporidium* sp. with 100% posterior probability. The genus *Helicosporidium* nests within the genus *Prototheca*, forming a clade with *P. wickerhamii* with 80% posterior probability. *Helicosporidium* is in red and other members of the *Prototheca*/*Auxenochlorella*/*Helicosporidium* clade are in purple.

DNA was extracted from fresh liver and kidney at the University of Florida Zoological Medicine and Wildlife Disease Laboratory using a DNeasy Kit (Qiagen, Valencia, California). Polymerase chain reaction for the protothecal partial *18S rRNA* gene was done as described.^9^ Products were electrophoresed in 1% agarose and purified using a commercial extraction kit (Qiagen). Direct sequencing was performed using a commercial kit and automated DNA sequencers (Applied Biosystems, Foster City, California). Primer sequences were trimmed off before further analysis. The sequence was submitted to GenBank (Accession No. OR913728).

Homologous nucleotide sequences from Chlorophyta *18S rRNA* genes were retrieved from GenBank and aligned using multiple alignment using fast Fourier transform (MAFFT).^5^ The *Helicosporidium* eDNA sequences were shorter, and ambiguities were added to their 3’ ends. *Watanabea acidotolerans* (GenBank MH827565), an alga in the Trebouxiophyceae outside the Chlorellales, was designated as the outgroup. Bayesian analyses of nucleotide alignments were performed using MrBayes 3.2.7a on the CIPRES server, with a general time reversible model, gamma distributed rate variation, and a proportion of invariant sites;^10^ the first 25% of 2 000 000 iterations were discarded as burn-in (Fig. 2d). The kingsnake sequence clustered within *Helicosporidium* sp. with 100% posterior probability. The genus *Helicosporidium* was found to nest within the genus *Prototheca*, forming a clade with *Prototheca wickerhamii* with 80% posterior probability.

The nesting of *Helicosporidium* within *Prototheca* is in agreement with other recent reports and has significant implications for evolution and nomenclature of the *Chlorellaceae*.^1,13^ The polyphyly of *Prototheca* implies that either *Helicosporidium* and *Auxenochlorella* should be subsumed into *Prototheca* or *Prototheca* should be divided into multiple genera. Some authors have recognized the longer genetic distance of *P. wickerhamii* from other members of the genus,^13,19^ and interspecific distances within *Prototheca* are greater than intergeneric differences in other Chlorophyta (Fig. 2d). Division of *Prototheca* into multiple genera would seem appropriate, but further data are needed to do so accurately. The *Prototheca*/*Helicosporidium*/*Auxenochlorella* clade contains all known algal pathogens of animals, and an understanding of their evolutionary adaptations for virulence is medically relevant.

The life cycle of *Helicosporidium* sp. has been partly elucidated under laboratory conditions using several species of insects.^2,3,4,7,8^ The mature cyst is the infectious form, and the route of infection is oral.^7,8^ Once in the insect’s midgut, the cysts rupture through dehiscence, releasing the ovoid and filamentous cells. The ovoid cells degrade, whereas the filamentous cells penetrate the intestinal epithelium and reach the hemocoel.^4^ In the hemolymph, the filamentous cells transform into vegetative cells that undergo several cycles of replication within a pellicle, eventually releasing daughter cells that replicate and differentiate into additional vegetative cells.^3^ A proportion of vegetative cells differentiate into cysts.^2^ It is unknown how the mature cysts are released from the host’s hemolymph and infect other insects, but death of the host with rupture of the exoskeleton has been suggested.^6,15^

The route of infection and life cycle in the kingsnake reported here are unknown. Infection may have occurred orally, and the filamentous cells could have ultimately entered the bloodstream via the larynx or the colon, which is supported by the fact that organisms were visualized at these sites; however, organisms were also observed in other organs without substantial microscopic lesions. Cysts, vegetative cells, and fragments of pellicles with curled borders were observed ultrastructurally, suggesting that *Helicosporidium* sp. was completing some of the steps of its life cycle in the kingsnake’s bloodstream. Finally, it is possible that the kingsnake was immunosuppressed. Criteria to evaluate immunosuppression in reptiles are poorly established, but temperature, season, husbandry conditions, and coinfections have been suggested as predisposing factors.^12^ The albinism of this animal also implies inbreeding, which results in selection for lack of immune diversity and decreased immune function.^11^ In this line, some bacteria were observed within the vessels of several organs, suggesting concurrent septicemia.

Histologic descriptions of *Helicosporidium* sp. in tissues are limited. Based on the findings of this case, cysts should be considered a diagnostic feature if the 3 parallel, stick-like ovoid cells are observed; the latter can be highlighted with a Gram stain. The organism’s wall has a similar tinctorial affinity for periodic acid-Schiff and Grocott-Gomori methenamine silver as *Prototheca* spp. and some fungi.^19^ However, some forms in our case were acid-fast, which may also be considered a differentiating diagnostic feature.

This case represents an example of spillover of a pathogen of invertebrates to a vertebrate host. *Helicosporidium* sp. may be expanding its host ratio, and this report will assist other pathologists in identifying this agent.
